# Gesundheitskompetenz im Zeitalter der Digitalisierung: Wo stehen wir?

**DOI:** 10.1007/s11553-021-00872-7

**Published:** 2021-07-01

**Authors:** Doris Schaeffer, Svea Gille

**Affiliations:** 1 Fakultät für Gesundheitswissenschaften, Interdisziplinäres Zentrum für Gesundheitskompetenzforschung (IZGK), Universität Bielefeld, Universitätsstr. 25, 33615 Bielefeld, Deutschland; 2 Hertie School, Friedrichstr. 180, 10117 Berlin, Deutschland

**Keywords:** Politikfeldentstehung, Digitale Transformation, Health Literacy, Gesundheitsinformation, Handlungsbedarf, Policy process, Digital transformation, Health literacy, Health information, Need for action

## Abstract

**Hintergrund:**

Mit der digitalen Transformation und dem Zuwachs an digitalen Gesundheitsinformationen steigen auch die Möglichkeiten der Gesundheitsförderung und Prävention. Gleichzeitig erhöhen sich auch die Anforderungen an den Umgang mit Gesundheitsinformationen und damit an die (digitale) Gesundheitskompetenz. Vorliegende Studien zeigen jedoch, dass ein Großteil der Bevölkerung in Deutschland nicht über ausreichende Gesundheitskompetenz verfügt und hier großer politischer Handlungsbedarf besteht.

**Ziel und Fragestellung:**

Der Beitrag geht der Frage nach, inwieweit es gelungen ist, Gesundheitskompetenz in Deutschland als Thema und als potenzielles neues Politikfeld zu etablieren.

**Material und Methoden:**

In Anlehnung an die Diskussion zur Ausreifung von Politikfeldern werden in dem Beitrag fünf Kriterien ((1.) Problemwahrnehmung und Agenda Setting, (2.) Konsens über Definition und Ziele, (3.) empirische Belege, (4.) Wille zum Handeln und Veränderungsbereitschaft, (5.) Ressourcenausstattung) betrachtet, anhand derer der Reifegrad von Gesundheitskompetenz als neues Politikfeld einzuschätzen versucht wird.

**Ergebnisse:**

Insgesamt zeigt sich, dass Gesundheitskompetenz in Deutschland eine rasante Themenkarriere durchlaufen hat und ein gesellschaftlich und politisch zunehmend relevantes Thema darstellt. Auch bei der Etablierung und Reifung als Politikfeld erfolgten in den letzten Jahren wichtige, auch international beachtete Schritte. Dabei wurde der Digitalisierung und der Verknüpfung beider bislang getrennt geführten Diskussionen und Entwicklungen jedoch zu wenig Beachtung geschenkt. Auch bis zur nachhaltigen Verankerung als Politikfeld sind noch etliche Herausforderungen zu bewältigen.

**Schlussfolgerung:**

Die Bedeutung von Gesundheitskompetenz ist im digitalen Zeitalter größer denn je. Die Bewältigung dieser Herausforderungen stellt daher eine zukünftig wichtige Aufgabe dar.

## Abstract (en)

**Background:**

With the digital transformation and the increase in digital health information, the opportunities for health promotion and disease prevention are also increasing. At the same time, the demands on dealing with health information and thus for (digital) health literacy are also increasing. However, existing studies show that a large part of the population in Germany is not adequately health literate, which points to a great need for political action.

**Objectives:**

This article therefore examines the extent to which health literacy has been successfully established as an issue and as a potential new policy area in Germany.

**Materials and methods:**

Following the discussion on policy analysis, the paper considers five criteria ((1.) problem perception and agenda-setting, (2.) consensus on definition and objectives, (3.) empirical evidence, (4.) willingness to act and change, (5.) level of resources) against which it attempts to assess the development of health literacy as a new policy area.

**Results:**

Overall, it can be shown that health literacy has undergone a rapid career as a topic in Germany and is becoming increasingly relevant both socially and politically. In recent years, important steps have been taken in establishing health literacy as a policy area, which have also attracted international attention. However, too little attention has been paid to digitalization and to linking the two discussions and developments, which have been conducted separately so far. To anchor health literacy sustainably as a policy field, several challenges have to be addressed.

**Conclusions:**

The importance of health literacy in the digital age is greater than ever. Meeting these challenges will therefore be an important task in the future.

Durch die digitale Transformation des Gesundheitswesens steigt der Bedarf an Gesundheitskompetenz. Aktuelle empirische Befunde zeigen jedoch, dass sich ein Großteil der Bevölkerung in Deutschland vor Schwierigkeiten im Umgang mit Gesundheitsinformationen gestellt sieht und weisen somit auf den bestehenden politischen Handlungsbedarf. In Anlehnung an die Diskussion zur Ausreifung von Politikfeldern geht der Beitrag der Frage nach, inwieweit es bislang gelungen ist, Gesundheitskompetenz auf der politischen Agenda in Deutschland zu etablieren und welche Herausforderungen bis zur nachhaltigen Verankerung als Politikfeld zukünftig zu bewältigen sind.

Es kommt heute fast einem Allgemeinplatz gleich: Allen Bereichen gesellschaftlichen Lebens hat die Digitalisierung einen enormen, temporeichen Modernisierungsschub beschert. Das gilt besonders für das Gesundheitswesen, sei es auf der Ebene der Prädiktion, der Diagnostik und Therapie, der Prävention und Gesundheitsförderung, der Arbeitsorganisation und Kooperation, des Wissenstransfers oder aber auf der Patientenebene. Dort bietet sie Möglichkeiten, den Umgang mit dem Versorgungssystem einfacher, transparenter, partizipativer und patientenfreundlicher zu gestalten, etwa die Navigation und Interaktion zu erleichtern (durch Suchmaschinen und Datenbanken, Onlineterminabsprachen, Videosprechstunde, elektronische Rezepte oder die elektronische Patientenakte [ePA]), Therapieregime zu unterstützen und das Selbstmanagement und -monitoring zu erleichtern (z. B. durch Apps, etwa zum Self-Tracking bzw. zur Messung/Kontrolle von Fitness- oder Vitalwerten). All diese Entwicklungen bringen viel an Entlastung, Annehmlichkeit, Autonomie und auch an Lebenszeitgewinn, Lebensqualität und Teilhabemöglichkeit mit sich.

Auch die Kommunikation und Information haben sich durch die Digitalisierung grundlegend verändert. Das Smartphone hat sich zu einem unverzichtbaren Begleiter des Alltags entwickelt und steht als Symbol dafür, dass mit der Digitalisierung viele neue, inzwischen längst selbstverständliche Kommunikations- und Informationsmöglichkeiten entstanden sind, die jeden dauernd – tags wie nachts, regional wie global – mit Information und Wissen versorgen. Information und Wissen sind dadurch heute prinzipiell jederzeit und an jedem Ort zugänglich, und die Menge an Informationen ist kaum noch überschaubar.^1^ Auch Fachwissen ist heute kein Spezialwissen mehr, das ausgewählten Kreisen vorbehalten ist. Es ist ebenfalls grenzenlos und prinzipiell allen (im Netz) zugänglich. Diese Entwicklung bringt – wie die Moderne generell – für die/den Einzelnen einen ungeahnten Zuwachs an neuen Optionen mit sich. Doch erhöhen sich zugleich die an sie/ihn gestellten Anforderungen und verlangen in allen Lebensbereichen Veränderungen, wodurch der Alltag anstrengender, anspruchsvoller und auch anfälliger für Überforderung wird [3].

Ähnliches gilt für den Zuwachs an *digitalen Gesundheitsinformationen*: er beschert ebenfalls viele neue Chancen und zugleich steigen mit ihm die Anforderungen an die (digitale) Gesundheitskompetenz der/des Einzelnen. Denn sie/er muss in der Lage sein, sich in einer täglich wachsenden Flut an Gesundheitsinformationen und einer unüberschaubaren Informationslandschaft zu orientieren, Information zu selektieren, filtern, zu dekodieren und einzuschätzen, auf die eigene Situation zu übertragen und sie zur Steuerung der eigenen Gesundheit zu nutzen – eine komplexe Aufgabe, zumal vermehrt interessengeleitete, falsche und auch manipulative Informationen im Umlauf sind, die vielfach auf Empörik, nicht aber seriöser Empirik basieren. Gerade in der digitalen Wissensgesellschaft wird solide und zugleich *kritische* Gesundheitskompetenz daher immer wichtiger, sowohl zur Prävention von Überstrapazierung und Überforderung als auch, um fragwürdige Entwicklungen zu verhindern, wie die Antiimpfkampagnen oder Verschwörungstheorien und Verleugnungen des Coronavirus SARS-CoV‑2 („severe acute respiratory syndrome coronavirus 2“) in den sozialen Medien deutlich machen. Doch zeigen die vorliegenden Studien zur Gesundheitskompetenz für Deutschland, dass nicht alle Menschen über diese Kompetenz verfügen [15, 34, 35]. Sie führen zugleich vor Augen, dass hier großer politischer Handlungsbedarf besteht.

Fragt sich, inwieweit es in den letzten Jahren gelungen ist, hier voranzukommen und Gesundheit und somit indirekt auch Gesundheitskompetenz in Deutschland als neues Politikfeld zu etablieren (Gesundheit in allen Politikbereichen/„health in all policies“ [HiAP]). Dieser Frage wird in dem folgenden Beitrag nachgegangen und dem Thema Digitalisierung dabei eine bedeutende Rolle beigemessen. Er orientiert sich an der Diskussion über die Ausreifung von Politikfeldern [12, 23, 29, 47, 48], die sich mit der Frage befasst, unter welchen Bedingungen neue Politikfelder zustande kommen – konkret: wie aus einem gesellschaftlichen Problem ein politisches Thema wird, welche Akteursnetzwerke sich herausbilden, um das Thema voranzubringen, wie sie es kommunizieren und bearbeiten und wie der Ausreifungsprozess verläuft. In der Literatur werden unterschiedliche Kriterien benannt, an denen der Grad der Ausreifung neuer Politikfelder eingeschätzt werden kann. In Anlehnung daran werden hier insgesamt fünf Punkte betrachtet: (1) Problemwahrnehmung und Agenda Setting, (2) Konsens über Definition und Ziele, (3) empirische Belege, (4) Wille zum Handeln und Veränderungsbereitschaft und (5) Ressourcenausstattung. Dabei wird gefragt, was sie im Fall des Themas Gesundheitskompetenz über den Reifegrad als Politikfeld aussagen und für die weitere Entwicklung lehren.

## Problemwahrnehmung und Agenda Setting

Begonnen wird mit der Frage, ob Gesundheitskompetenz hierzulande als gesellschaftlich relevantes Problem wahrgenommen wird und einen Platz auf der politischen Agenda einnimmt. Kristine Sørensen – bekannt durch die häufig verwendete Definition von Gesundheitskompetenz – hält dies international für entschieden: „Health literacy has come to stay, and developments occur fast“ [42], so ihre Einschätzung, nach der Health Literacy bzw. Gesundheitskompetenz international fest als Thema und auch als Politikfeld etabliert ist. Eine ganze Reihe an Dokumenten der Weltgesundheitsorganisation (WHO) belegt dies (z. B. [51–54]).

Es wäre wünschenswert, wenn dies auch für Deutschland schon mit fester Stimme behauptet werden könnte, zumal hier mit großer Geschwindigkeit vieles erreicht wurde. Zwar wurde in Deutschland erst vor ca. 10 Jahren mit der Diskussion über Gesundheitskompetenz begonnen und damit deutlich später als in vielen anderen Ländern – besonders als in den USA, wo das Thema seit mehr als 40 Jahren bearbeitet wird. Aber seither wurde in Deutschland mit großen Schritten aufzuholen versucht. Das zeigt sich u. a. darin, dass das Thema inzwischen politisch präsent ist. So war Gesundheitskompetenz 2016 ein Themenschwerpunkt des Treffens der fünf deutschsprachigen Gesundheitsminister. 2017 hat das Bundesministerium für Gesundheit (BMG) die Allianz für Gesundheitskompetenz gegründet mit dem Ziel, Projekte zur Förderung von Gesundheitskompetenz zu initiieren. 2018 wurde die Förderung von Gesundheitskompetenz in den Koalitionsvertrag der Bundesregierung aufgenommen und fand in den Beschluss der 91. Gesundheitsministerkonferenz Eingang. Ebenfalls seit 2018 existiert in Deutschland ein Nationaler Aktionsplan Gesundheitskompetenz (NAP), wie es ihn weltweit nur in ungefähr 10 Ländern gibt [36]. In den jüngst verabschiedeten Digitalisierungsgesetzen ist die Förderung von (digitaler) Gesundheitskompetenz sogar explizit als Aufgabe der Krankenkassen aufgenommen worden (DVG, § 20k SGB V).

Exemplarisch verdeutlicht dies den politischen Bedeutungsgewinn des Themas. Er wird auch daran sichtbar, dass inzwischen etliche politische Akteure und Ministerien begonnen haben, sich für die Förderung von Gesundheitskompetenz zu engagieren. Anzuführen sind neben dem BMG, in dem inzwischen ein eigenes Referat „Nachhaltigkeit und Gesundheitskompetenz“ existiert, das Bundesministerium der Justiz und für Verbraucherschutz (BMJV), das Bundesministerium für Ernährung und Landwirtschaft (BMEL) und das Bundesministerium für Bildung und Forschung (BMBF).

Auch auf der Landesebene regt sich vieles. Etliche Landesvereinigungen für Gesundheit haben Gesundheitskompetenz als Thema aufgegriffen und teilweise länderspezifische Strategien (oft abgeleitet aus dem NAP) entwickelt. Stellvertretend für andere sei Hamburg und der dort eigens entwickelte Strategie- und Maßnahmenplan erwähnt [41].

Entstanden sind zudem eine Reihe wichtiger Akteursnetzwerke und Interessengruppen zum Thema Gesundheitskompetenz, die für die Entstehung neuer Politikfelder wichtig sind. Erinnert sei nochmals an die 2017 gegründete Allianz für Gesundheitskompetenz, ebenso an den NAP mit seinem Netzwerk. Zu erwähnen sind auch das 2019 gegründete Deutsche Netzwerk Gesundheitskompetenz [33], die Arbeitsgruppe Gesundheitskompetenz im Deutschen Netzwerk für Versorgungsforschung [10] oder der Fachbereich Patienteninformation und -beteiligung im Deutschen Netzwerk Evidenzbasierte Medizin [21]. Jüngst hat sich zudem eine Allianz Gesundheitskompetenz und Schule gegründet [25]. Daneben sind etliche Forschungsnetzwerke entstanden. Exemplarisch sei hier das unter dem Dach der WHO Europa gegründete Action Network on Measuring Population and Organizational Health Literacy erwähnt, das als Rahmen für eine regelmäßige Erhebung populations- und organisationsspezifischer Health-Literacy-Daten dient, und an dem auch Deutschland beteiligt ist [9]. Zugleich haben sich etliche themenspezifische und regionale Netzwerke gebildet. Einige sind noch in der Findungs- und Konsolidierungsphase, andere haben bereits ein Programm. Fast alle verfolgen sie als Aufgabe, Aktivitäten zur Stärkung der Gesundheitskompetenz anzuregen, den Austausch zu stimulieren und das Thema Gesundheitskompetenz voranzubringen.

Dennoch bleibt als Aufgabe, die Problemwahrnehmung weiter zu schärfen und dafür zu sorgen, dass das Thema Gesundheitskompetenz einen *stabilen* und nachhaltigen Platz auf der Agenda erhält. Sehr wichtig ist unter Agenda-Setting-Aspekten außerdem, *bislang unabhängig voneinander geführte Diskussionen besser zu verknüpfen*, womit das Thema Digitalisierung angesprochen ist.

Über beide Themen wird in Deutschland aktuell viel, aber weitgehend getrennt voneinander diskutiert. In der Gesundheitskompetenzdiskussion ist das Thema Digitalisierung bislang eher marginal beachtet worden und umgekehrt ist es in der Digitalisierungsdiskussion. Dort stehen eher die technischen Optionen und der Modernisierungsdruck im Bereich Digitalisierung in Deutschland im Vordergrund. Themen wie Gesundheitskompetenz und auch Nutzerorientierung werden dort erst langsam als bedeutsam erkannt. Mit der COVID-19-Pandemie („coronavirus disease 2019“) beginnt sich das zu verändern, aber noch nicht weitreichend genug. Angesichts der aktuell umzusetzenden Digitalisierungsgesetze ist das besonders zu betonen, denn mit den Gesetzen^2^ erhöht sich der Handlungs- und Veränderungsdruck in der Praxis. Soll er in die richtigen Bahnen gelangen, *müssen* beide Diskussionen zusammenfließen – allein um die mit der Umsetzung der Gesetze in der Praxis anstehenden Aufgaben auf der Akteursebene zielkonform bewältigen zu können. Zugleich geht die Umsetzung mit einem enormen Bedarf an Information und Know-how einher. Um ihn aufzufangen sind Aufgaben anzugehen, für die es noch weitgehend an tragfähigen Konzepten und Kompetenzen fehlt.

## Konsens über Definition sowie Ziele und Art der Problembearbeitung

Entscheidend für die Etablierung als Politikfeld ist ein weitgehender *Konsens über Definition sowie Ziele und Art der Problembearbeitung *[23, 29, 31]. Auf das hier zur Diskussion stehende Thema Gesundheitskompetenz bezogen bedeutet dies Einverständnis darüber, was unter Gesundheitskompetenz und digitaler Gesundheitskompetenz zu verstehen ist und wie Verbesserungsmaßnahmen erfolgen können.

Über dieses Thema wurde in Deutschland eine lebendige Diskussion geführt – zu Beginn eher *euphorisiert*, weil der englische Begriff Health Literacy, der zunächst noch nicht übersetzt verwendet wurde, so neu, so schillernd und vielversprechend war und daher überall faszinierte; später eher ernüchtert, weil die sich rasch durchsetzende Übersetzung von Health Literacy mit Gesundheitskompetenz Missverständnisse erzeugte und nicht überall auf Akzeptanz stieß. Die Diskussion war daher nicht immer einfach, hat aber im Ergebnis dazu geführt, dass über wichtige Eckpunkte hierzulande relative Einigkeit herrscht. So wird Gesundheitskompetenz meist in Anlehnung an den Europäischen Health Literacy Survey (HLS-EU) als das Wissen, die Motivation und Fähigkeit dazu definiert, gesundheitsrelevante Information in unterschiedlicher Form finden, verstehen, beurteilen und anwenden zu können, um im Alltag tragfähige Entscheidungen zur Gesundheitserhaltung und -förderung treffen zu können [43]. Ebenso herrscht weitgehend Konsens darüber, einem *„relationalen“* Verständnis von Gesundheitskompetenz zu folgen, nach dem Gesundheitskompetenz sowohl durch persönliche Fähigkeiten geprägt wird wie auch durch die umgebenden (strukturellen) Bedingungen [26]. Zweierlei ist daran folgenreich:

- Nach diesem Verständnis sollten auch Interventionsmaßnahmen *beidseitig* ansetzen: sowohl auf die Stärkung persönlicher Fähigkeiten wie auch auf die Veränderung der Lebensumwelten und Strukturen zielen, also beispielsweise auch auf Veränderung des Gesundheitssystems hin zu einem nutzerfreundlichen und gesundheitskompetenten System und ebensolchen Organisationen, in denen der Umgang mit digitalen Optionen selbstverständlich ist.
- Die Definition schließt *digitale *Gesundheitsinformation ein, denn laut Definition geht es um gesundheitsrelevante Informationen *in unterschiedlicher Form*, ergo auch in digitaler Form [4, 10]; ebenso um die zum Umgang mit ihnen erforderlichen Kompetenzen. Eine neue Definition – wie sie in Deutschland zuweilen gefordert wird – ist daher nicht nötig; die bisherige reicht als Basis. Eine neue Definitionsdiskussion würde zudem Ressourcen binden, die aktuell dringend an anderer Stelle benötigt werden und ungewollt kontraproduktive Schulenbildungen verstärken.

Als *Zwischenfazit* bleibt hier somit festzuhalten, dass bei der Konsensherstellung über die Definition und die Ziele der Bearbeitung viel erreicht wurde, aber ansteht, die Verknüpfung von Gesundheitskompetenz mit anderen „literacies“ und besonders mit digitaler Gesundheitskompetenz zu festigen und Schulenbildungen zu vermeiden.

## Empirische Belege: Forschung

Die Etablierung eines stabilen Platzes auf der politischen Agenda oder eines Politikfelds setzt ausreichende *empirische Belege* voraus, so Whitehead [48]. Sie hält die Existenz solcher Belege sogar für entscheidend.

Die deutsche Diskussion ist ein guter Beleg für diese These. Schon seit ungefähr 2003 wurde in Deutschland über das Gesundheitskompetenzkonzept diskutiert [1, 17], doch erst die ab ca. 2014 erscheinenden empirischen Befunde setzten den Initialfunken für eine breitenwirksame Debatte. Erst also die Messbarkeit von Gesundheitskompetenz hat dazu geführt, dem Thema in der Politik eine größere Beachtung zu schenken. Dazu haben außerdem die ersten Studienergebnisse beigetragen. Danach verfügen mehr als 40 Mio. Menschen in Deutschland – mehr als die Hälfte der Bevölkerung – über eine geringe Gesundheitskompetenz [15, 35, 55]. Diese Zahlen haben irritiert und beunruhigt, weil sie unübersehbar gemacht haben, dass geringe Gesundheitskompetenz auch in Deutschland ein Problem darstellt. Dies hat sich sogar verschärft, wie die neuesten Befunde belegen. Denn der Anteil geringer Gesundheitskompetenz hat sich von 54,3 % im Jahr 2014 auf 64,2 % erhöht [14]. Insgesamt hat sich die Gesundheitskompetenz in Deutschland also verschlechtert, wie eine weitere Studie bestätigt [34]. Zugleich wurden damit empirische Belege erbracht, die die Bedeutung des Themas sichtbar machen. Es sind allerdings *erste* Belege. Ausreichende Belege setzen eine breite empirische Datenbasis und ausreichende Forschung voraus. Wie steht es darum?

Auch hier wurde in den letzten fünf bis sechs Jahren ein bemerkenswerter Fortschritt erreicht. Denn in Kürze ist eine engagierte wissenschaftliche Community entstanden, die wichtige Studien auf den Weg gebracht hat (Übersicht [10]). Aber auch in der Forschung bestehen noch etliche Herausforderungen – speziell, um den Anschluss an die internationale Entwicklung zu finden [10]. Zwar existieren in Deutschland inzwischen empirische Daten, doch bedürfen sie – wie eben angedeutet wurde – der Ergänzung und Erweiterung. Zudem steht an, die Datenbasis zu vertiefen und zu einer *regelmäßigen* Messung der Gesundheitskompetenz der Bevölkerung zu kommen [9, 36]. Auch über spezifische Zielgruppen, z. B. *vulnerable Bevölkerungsgruppen*, ist das empirische Wissen noch gering. Das gilt auch für Nutzergruppen, die das Gesundheitswesen besonders häufig frequentieren: für Menschen mit chronischer Krankheit und ältere Menschen, doch ebenso für Menschen mit niedrigem Sozialstatus und mit geringer Bildung. Sie haben, wie die Daten zeigen, erhebliche Schwierigkeiten im Umgang mit (digitalen) Gesundheitsinformationen. Um für sie passgenaue und evidenzbasierte Interventionskonzepte, wie z. B. digitale Gesundheitsanwendungen zur Stärkung der Gesundheitskompetenz zu entwickeln, ist tiefergehendes empirisches Wissen unverzichtbar. Ähnliches lässt sich auch für Kinder und Jugendliche bzw. junge Erwachsene oder aber die Instrumentenentwicklung festhalten, denn die vorhandenen Messinstrumente sind nicht für alle Bevölkerungsgruppen geeignet (z. B. [6]).

Auch zur *digitalen *Gesundheitskompetenz fehlt es noch an empirischem Wissen. Erst seit kurzem liegen dazu empirische Befunde vor [8, 18, 34]. Doch insgesamt mangelt es an ausreichenden Erkenntnissen über das Ausmaß digitaler Gesundheitskompetenz in der Bevölkerung und bei einzelnen Bevölkerungsgruppen sowie den vorhandenen Unterstützungsbedarf. Noch weniger ist darüber bekannt, welche Konsequenzen die Digitalisierung auf notwendige Veränderung der Lebensumwelten und Strukturen hinsichtlich gesundheitskompetenter Systeme und Organisationen haben muss.

Vornehmlich zum *digitalen Gesundheitsinformationsverhalten* liegen bereits seit längerem Daten vor [20, 22, 30, 45, 56]. Aber auch dies sind *erste empirische Daten*, die der Vertiefung bedürfen, zumal sie teilweise widersprüchlich sind und auf unterschiedlichen methodischen Vorgehensweisen basieren. Dennoch: diese Studien zeigen, dass die deutsche Bevölkerung nicht so medienaffin ist, wie die anderer Länder, wie sich auch im Digitalisierungsgrad der Bevölkerung widerspiegelt. Dem Digital Economy and Society Index (DESI) zufolge lag Deutschland 2020 nur knapp über dem EU-Durchschnitt [11]. Gleichzeitig hat die Nutzung digitaler Information(smedien) auch in Deutschland an Bedeutung gewonnen – insbesondere bei Menschen im jüngeren und mittleren Lebensalter und dann, wenn ein konkretes Krankheitsproblem vorliegt [20, 22, 45, 56]. Oft wird dann Dr. Google zu Rate gezogen, was Ärzt:innen nicht immer schätzen [5, 28, 38]. Insgesamt steigt die Bedeutung des Internets für die Suche nach krankheitsrelevanter Information; es steht inzwischen an zweiter Stelle der präferierten Informationsquellen [2, 14]. Die Nutzung anderer digitaler Informationsangebote ist dagegen in Deutschland noch übersichtlich. So werden etwa Gesundheits-Apps nur von 20–30 % der Bevölkerung in Deutschland genutzt [20, 34], obschon sie hier – international einmalig – ärztlich verordnet und von den Krankenkassen erstattet werden können und auch zur Stärkung der Gesundheitskompetenz beitragen dürften.

Wenig empirisches Wissen liegt bislang über *die Verarbeitung digitaler Informationen* in Deutschland vor. Dies genauer zu erforschen ist jedoch wichtig, denn die *bloße Verfügbarkeit von Information macht noch nicht klüger*. Eher gegenteilig, mit der Vielfalt und Zersplitterung an Information und der Zunahme an fragwürdiger digitaler Information nimmt auch die Orientierungslosigkeit zu und steigen die Anforderungen an den Umgang mit Information. Besonders die Beurteilung der Vertrauenswürdigkeit digitaler Gesundheitsinformationen fällt der Bevölkerung schwer [34]. Vermutlich stoßen integrierte Gesundheitsportale mit verlässlichen und gebündelten Informationen deshalb bei vielen Nutzer:innen auf Zustimmung [38]. Zugleich hat die digitale Informationsvielfalt einen anderen negativen Effekt: Sie verstärkt die digitale Spaltung [7] – eine Entwicklung, die international bereits hohe Beachtung erhält – in Deutschland bislang zu wenig.

Dieser kurze Abriss deutet an, dass die Forschung zur Gesundheitskompetenz zwar zunimmt, aber auch hier noch etliche Aufgaben bestehen. Sie anzugehen ist kein „nice to do“, sondern eine essenzielle Voraussetzung für die Entwicklung abgesicherter, tragfähiger Interventionen, die auf die gegebenen Informationsvoraussetzungen zugeschnitten sind und nicht über die Köpfe hinweggehen und auch für die Beförderung der Politikfeldentwicklung.

### Interventionsentwicklung und -forschung

Dies führt zu der Frage, wie es um die *Interventionsentwicklung* bestellt ist. Auch dazu sind etliche Projekte und Initiativen entstanden. So wurden beispielsweise eine Strategie zur Mundgesundheitskompetenz erarbeitet [19], ebenso Material- und Methodensammlungen (z. B. [39]), Kurzinformationen oder evidenzbasierte Materialien für den Schulunterricht [44].

Auch zur Förderung der *digitalen* Gesundheitskompetenz existieren erste Projekte. Exemplarisch sei hier auf eine Orientierungshilfe zum Umgang mit digitalen Gesundheitsinformationen [16] hingewiesen. Auch die entstandenen Portale, wie etwa das 2020 vom Bundesministerium initiierte Nationale Gesundheitsportal (www.gesund.bund.de), sind hier zu erwähnen. Meist widmen sich die Projekte der Förderung der persönlichen Gesundheitskompetenz und der Verbesserung digitaler Information (z. B. durch Erklärfilme). Kontext- oder lebensumweltbezogene Interventionen, wie sie dem relationalen Verständnis von Gesundheitskompetenz entsprechen, sind noch rar. Vielfach konzentrieren sich die Bemühungen zudem noch auf die Erschließung und Übertragung andernorts erarbeiteter Maßnahmen und Konzepte, die oft, aber nicht immer passungsfähig mit den hiesigen Bedingungen sind. Exemplarisch deutet dies auf den hier bestehenden Handlungsbedarf, der mit der Coronapandemie und den jetzt umzusetzenden Digitalisierungsgesetzen noch einmal größer geworden ist. Darauf antwortende Interventionskonzepte zu erarbeiten, ist daher dringender denn je, zumal solche Situationen – großer Handlungsdruck ohne verfügbare Konzepte – immer anfällig für unerwünschte Entwicklungen sind, u. a. dafür, dass rasch alter Wein in neue Schläuche gegossen wird – ein Phänomen, das auch im Bereich Gesundheitskompetenz vertraut ist [24].

Langsam nimmt auch die Interventionsforschung zur Gesundheitskompetenz zu; doch faktisch ist sie noch eher im Begriff des Werdens. Damit stehen wir in Deutschland nicht allein: Auch international ist die Interventionsforschung noch entwicklungsbedürftig [24, 46]. Hinzu kommt, dass Interventionsentwicklung ein langwieriges Unterfangen ist: Seriös betrieben, dauert die wissenschaftlich gesicherte Entwicklung, Testung, Überarbeitung, Implementation und Wirksamkeitsprüfung aufgrund der immer differenzierteren Anforderungen viele Jahre und ist sehr ressourcenintensiv. Weil der soziale Wandel genau gegenläufig immer temporeicher wird, ist eine Intervention bis sie als gesichert bezeichnet werden kann, oft schon veraltet. Das gilt speziell für Interventionen zur Stärkung der digitalen Gesundheitskompetenz. Hier wird neu nachzudenken sein.

Festhalten lässt sich also, dass die Interventionsentwicklung begonnen hat, aber gerade hier noch viele Herausforderungen anzugehen sind [10, 37].

## Wille zum Handeln und Veränderungsbereitschaft

Wichtig für die Etablierung eines Politikfelds ist außerdem, dass *Wille zum Handeln* und *Veränderungsbereitschaft *existieren. Wie sieht es damit aus: Ist es gelungen, im Gesundheitssystem, seinen Organisationen, Verbänden und Professionen ausreichend Verständnis und Handlungsbereitschaft für die Stärkung von Gesundheitskompetenz zu erzeugen?

Auch hier ist das Wasserglas halbvoll und halbleer. Das Interesse am Thema Gesundheitskompetenz ist deutlich größer als vor 5 bis 6 Jahren, wenngleich dies aktuell durch die Coronapandemie überlagert wird. Zu beobachten ist aber, dass viele der großen Verbände und Organisationen im Gesundheitswesen inzwischen sensibilisiert sind. Einige sind mittlerweile sogar dabei, systematische Strategien zur Förderung der Gesundheitskompetenz in ihrem Wirkungsfeld zu entwickeln, andere sind abwartend, manche auch skeptisch.

Auf organisatorischer Ebene ist die Situation ähnlich, wie die Diskussion über „gesundheitskompetente Organisationen“ exemplarisch zeigt [32, 37]. Aktuell wird dieser Ansatz in Deutschland favorisiert, u. a. weil er ermöglicht, nicht die Menschen zum Problem zu erheben, sondern die an ihnen manifest werdenden veränderungsbedürftigen Strukturen. Die Umsetzung steht jedoch noch am Anfang. Zwar liegen international bereits erprobte Konzepte und Instrumente vor (z. B. [27]), aber sie müssen vielfach noch an die hiesigen Bedingungen angepasst werden. Überwiegend wurden sie für den Krankenhausbereich entwickelt, sodass außerdem ansteht, sie auf andere Organisationen und Bereiche zu übertragen. Zudem ist auch hier erforderlich, die Forschung zu intensivieren und die Kapazitätsentwicklung anzustoßen. Wie wichtig dies ist, wird u. a. daran deutlich, dass in Deutschland noch kein entsprechender Modellversuch existiert.

Das Thema Digitalisierung spielt auch in dieser Debatte bislang so gut wie keine Rolle. Nicht zuletzt die Coronapandemie hat gezeigt, dass dies der Veränderung bedarf und beide Themen zusammen angegangen werden sollten, denn sonst verlaufen Veränderungsprozesse in den Einrichtungen parallel und stehen dann in Gefahr, nicht zusammen zu fließen.

Es bleibt die *Gesundheitsprofessionen und -berufe *zu betrachten, denn sie haben nach wie vor hohe Bedeutung für die Stärkung der Gesundheitskompetenz. Das gilt speziell für die Ärzt:innen, die bis heute die wichtigste Informationsquelle darstellen [2, 14].

Fragt man danach, ob bei ihnen ausreichend Verständnis, Kompetenz und Kapazität zur Förderung von Gesundheitskompetenz herrscht, lässt sich ähnliches wie zuvor konstatieren: Bei vielen Gesundheitsberufen besteht Interesse am Thema. Ob auch der Handlungswille wächst, lässt sich nur schwer einschätzen. Zwar sind viele Beispiele anzutreffen, die zeigen, dass sich einzelne Ärzt:innen, Pflegende oder Angehörige der Therapieberufe engagiert um patientengerechte Information und Edukation bemühen. Doch stoßen sie dabei auf strukturelle (besonders zeitliche und finanzielle) und qualifikatorische Hürden und nicht selten bremsen diese mit der Zeit ihre Handlungsbereitschaft. In vielen Einrichtungen besteht zudem großer Problemdruck, der während der Coronapandemie noch einmal deutlich gestiegen ist. Auch das bremst individuell und institutionell aktuell die Innovationsbereitschaft bei den Gesundheitsprofessionen. Es verdeutlicht überdies, dass die Förderung von Gesundheitskompetenz vielfach begleitende Strukturinnovationen erfordert.

Beachtliche Fortschritte sind bei dem Thema Kompetenz- und Kapazitätsentwicklung zu verzeichnen. So nimmt die Zahl der Studiengänge oder Studiengangschwerpunkte zur Gesundheitskommunikation zu und gleichzeitig soll das Thema Kommunikation/Information in die Curricula der Medizinerausbildung Einkehr halten [13], ebenso in die Rahmenlehrpläne der Pflegeberufe und teilweise auch in Fortbildungscurricula. Doch bestehen auch hier Herausforderungen. So findet der Begriff Gesundheitskompetenz in den Rahmenlehrplänen für die Pflege nur ganze 10-mal Erwähnung – wenig, aber immerhin.^3^ Digitalisierung taucht dagegen nur einmal auf. Pflegende sind somit weder auf die mit der Digitalisierung einhergehenden Anforderungen vorbereitet noch auf die Förderung (digitaler) Gesundheitskompetenz und das dürfte die Umsetzung des DVPMG enorm behindern, wie sich schon jetzt abzeichnet. Ähnliches gilt auch für die anderen Gesundheitsprofessionen/-berufe.

Zugleich zeigt dies, dass das Thema *digitale *Gesundheitskompetenz intensiverer Beachtung *in der Aus‑, Fort- und Weiterbildung der Gesundheitsberufe *bedarf, denn durch die aktuell im Zuge der Coronapandemie rasch voranschreitende Digitalisierung und die dazu auf den Weg gebrachten Gesetzesinitiativen wird die Kompetenzentwicklung im Umgang mit digitaler Gesundheitsinformation auch für die Gesundheitsprofessionen immer wichtiger.

Allerdings liegt bislang mangels Studien wenig fundiertes Wissen über den konkret bestehenden Informationsbedarf vor – weder bei den Versorger:innen, also den Gesundheitsprofessionen und -berufen, noch bei den Patient:innen und auch nicht bei den Kassen, die jetzt gesetzlich mit der Information betraut sind, aber ebenso wie die Gesundheitsprofessionen von dieser Aufgabe überfordert sein dürften. Aus Analysen zum Fortbildungsbedarf anderer Berufsgruppen lässt sich schließen, dass der Kompetenz- und Kapazitätsaufbau in Sachen Digitalisierung im Gesundheitssystem ebenfalls groß ist.

Somit ist auch hier als *Zwischenfazit* festzuhalten, dass die Sensibilisierung für die Förderung von Gesundheitskompetenz im Gesundheitssystem gestiegen ist und auch das Problemverständnis deutlich zugenommen hat. Gleichwohl bleibt als Herausforderung, die Handlungsveränderungsbereitschaft weiter anzuregen und zu intensivieren – dies auf allen Ebenen. Dazu ist auch, aber nicht allein weitere Überzeugungs- und Motivationsarbeit notwendig. Zugleich ist zu bedenken, dass Veränderungen oft langjährig verankerte professionelle und institutionelle Routinen berühren und daher immer (unbequeme) Umstellungsprozesse erfordern.

Erforderlich ist zudem auch hier, die Diskurse über Gesundheitskompetenz und Digitalisierung enger zusammenzuführen und die Voraussetzungen dafür zu schaffen, dass künftig ausreichend qualifizierte Expert:innen für die Förderung der (digitalen) Gesundheitskompetenz zur Verfügung stehen und es möglich wird, sich schrittweise der Vision eines gesundheitskompetenten Gesundheitssystems zu nähern [42], in dem der Umgang mit digitalen Optionen selbstverständlich ist.

## Ressourcensituation

Der Reifegrad als Politikfeld lässt sich u. a. daran bemessen, inwieweit ein nachhaltiger Ressourcenfluss für die Bearbeitung des jeweiligen Problems gewährleistet ist [29]. Deshalb wird als letztes die Ressourcensituation thematisiert. Sie lässt sich relativ kurz abhandeln, denn an ausreichenden und nachhaltigen Ressourcen für die Förderung von Gesundheitskompetenz hapert es weitgehend. Überwiegend werden bislang zeitlich befristete Einzelprojekte gefördert und fehlt es an koordinierten, systematischen Förderprogrammen und nachhaltigen Ressourcen. Das hemmt gegenwärtig sowohl die Interventionsentwicklung in der Praxis und die Forschung zur Gesundheitskompetenz als auch die Schaffung von nachhaltigen Strukturen für Gesundheitskompetenz und die dringend benötigte Aus‑, Fort- und Weiterbildung von Fachkräften. Anders sieht es im Bereich der Digitalisierung aus, dort stehen weitaus mehr Ressourcen zur Verfügung, doch – wie erwähnt – werden sie nur dann wirklich fruchten, wenn sie auch auf der Ebene der umsetzenden Akteure durch hinreichende Kompetenzförderung begleitet werden.

## Fazit

Gezeigt werden sollte, dass Gesundheitskompetenz ein gesellschaftlich zunehmend relevantes Thema darstellt, das durchaus das Potenzial hat, ein neues Politikfeld zu werden – davon ist nach der rasanten Themenkarriere in den letzten 5 bis 6 Jahren auszugehen. Befördert wird dies durch die digitale Transformation, die in der hiesigen Gesundheitskompetenzdiskussion allerdings noch zu geringe Aufmerksamkeit erfährt. Das gilt auch für die digitale Gesundheitskompetenz, die Teil von Gesundheitskompetenz ist und angesichts in Deutschland bestehenden Aufholbedarfs bei der Digitalisierung wie auch der aktuellen Coronapandemie überaus bedeutsam ist [40]. Denn einmal mehr hat die durch die Pandemie ausgelöste Krise gezeigt, wie wichtig Gesundheitskompetenz ist: Der Kampf gegen die Ausweitung der Pandemie und die Prävention von SARS-CoV-2-Infektionen sind ganz entscheidend auf einen kompetenten Umgang mit Gesundheitsinformationen angewiesen, die zu einem großen Teil in digitaler Form zugänglich sind und verbreitet werden. Dass hier gerade in Deutschland ein Problem besteht, belegen die wenigen dazu vorliegenden Studien, denn sie zeigen, dass sich die digitale Gesundheitskompetenz in Deutschland eher bescheiden ausnimmt. Die Förderung von Gesundheitskompetenz kann und sollte daher nicht ohne digitale Gesundheitskompetenz gedacht werden und ist im digitalen Zeitalter wichtiger denn je.

Der bislang erreichte Stand bei der Politikfeldetablierung hält damit nicht Schritt. Zwar ist es in Deutschland gelungen, gewissermaßen als Späteinsteiger in die Diskussion über Gesundheitskompetenz, in den letzten Jahren mit hohem Tempo und großem zivilgesellschaftlichen Engagement voranzukommen und dabei auch international beachtete (politische) Schritte zu tun. Man denke allein daran, dass in Deutschland – international einmalig – bereits ein Nationaler Aktionsplan zur Förderung der Gesundheitskompetenz *und* eine Allianz wichtiger Akteure aus dem Gesundheitssystem existieren und weitere wichtige Akteursnetzwerke entstanden sind, wie sie für die Etablierung als Politikfeld als wichtig erachtet werden. Allein dies kennzeichnet exemplarisch den erreichten Progress. Doch stehen bis zur nachhaltigen Etablierung als Politikfeld noch etliche Herausforderungen an. Das gilt auch für die digitale Gesundheitskompetenz – trotz der verabschiedeten Digitalisierungsgesetze, in denen das Thema digitale Gesundheitskompetenz erstmals gesetzlich verankert wurde. Die zurückliegenden Ausführungen zeigen aber hoffentlich, dass es lohnt und wichtig ist, diese Herausforderungen anzugehen.

## Fazit für die Praxis

- Die Förderung von Gesundheitskompetenz hat sich in den letzten Jahren zu einer wichtigen politischen und praktischen Aufgabe entwickelt, die für die Prävention und Gesundheitsförderung hohe Bedeutung hat.
- Durch die voranschreitende Digitalisierung erlangt sie noch einmal größeren Stellenwert. Denn mit ihr nimmt die Zahl und Vielfalt an gesundheitsrelevanten Informationen exponentiell zu und steigen zugleich die Anforderungen an den Umgang mit Informationen und damit an die Gesundheitskompetenz – eine Entwicklung, die auf politischer und praktischer Ebene nach größerer Handlungsbereitschaft ruft.
- Das gilt sowohl für die Etablierung als neues Politikfeld als auch als Handlungsfeld in der Praxis und hier im Bereich der Prävention und Gesundheitsförderung wie auch im Gesundheitssystem.

## Footnotes

1 Ein Beispiel: Im Mai 2021 waren in der Internetenzyklopädie Wikipedia über 56 Mio. Artikel verfügbar; davon 2,6 Mio. Artikel in deutscher Sprache [49]. Auch die Anzahl an Apps steigt kontinuierlich.

2 Zur Digitalisierung im Gesundheitswesen sind jüngst drei Gesetze verabschiedet worden. Dazu gehören (1) das Gesetz für eine bessere Versorgung durch Digitalisierung und Innovation (DVG), das u. a. eine Versorgung mit digitalen Gesundheitsanwendungen, wie etwa Gesundheits-Apps, den Ausbau telemedizinischer Angebote und die Einführung einer elektronischen Patientenakte vorsieht; (2) das Gesetz zum Schutz elektronischer Patientendaten in der Telematikinfrastruktur (PDSG) und (3) das Gesetz zur digitalen Modernisierung von Versorgung und Pflege (DVPMG), mit dem u. a. die Integration der Pflege in die Telematikinfrastruktur und die Einführung digitaler Pflegeanwendungen, ebenso eine Erweiterung der elektronischen Patientenakte und elektronischer Verschreibungen beabsichtigt wird.

3 Ähnlich ist es beispielsweise in der Physiotherapie [50].
